# A Review of the Recent Advances Made with SIRT6 and its Implications on Aging Related Processes, Major Human Diseases, and Possible Therapeutic Targets

**DOI:** 10.3390/biom8030044

**Published:** 2018-06-29

**Authors:** Rubayat Islam Khan, Saif Shahriar Rahman Nirzhor, Raushanara Akter

**Affiliations:** Department of Pharmacy, BRAC University, 1212 Dhaka, Bangladesh; rubayat.khan@bracu.ac.bd (R.I.K.); saif.rahman@bracu.ac.bd (S.S.R.N.)

**Keywords:** SIRT6, diabetes, gluconeogenesis, cancer, aging, heart disease, pharmacological SIRT6 inhibitor, cardiac hypertrophy, tumorigenesis, neurodegeneration, neurodegenerative diseases, Alzheimer’s Disease

## Abstract

Sirtuin 6 (SIRT6) is a nicotinamide adenine dinucleotide^+^ (NAD^+^) dependent enzyme and stress response protein that has sparked the curiosity of many researchers in different branches of the biomedical sciences. A unique member of the known Sirtuin family, SIRT6 has several different functions in multiple different molecular pathways related to DNA repair, glycolysis, gluconeogenesis, tumorigenesis, neurodegeneration, cardiac hypertrophic responses, and more. Only in recent times, however, did the potential usefulness of SIRT6 come to light as we learned more about its biochemical activity, regulation, biological roles, and structure Frye (2000). Even until very recently, SIRT6 was known more for chromatin signaling but, being a nascent topic of study, more information has been ascertained and its potential involvement in major human diseases including diabetes, cancer, neurodegenerative diseases, and heart disease. It is pivotal to explore the mechanistic workings of SIRT6 since future research may hold the key to engendering strategies involving SIRT6 that may have significant implications for human health and expand upon possible treatment options. In this review, we are primarily concerned with exploring the latest advances in understanding SIRT6 and how it can alter the course of several life-threatening diseases such as processes related to aging, cancer, neurodegenerative diseases, heart disease, and diabetes (SIRT6 has also shown to be involved in liver disease, inflammation, and bone-related issues) and any recent promising pharmacological investigations or potential therapeutics that are of interest.

## 1. Introduction

Sirtuins are a family of enzymes, which are nicotinamide adenine dinucleotide^+^ (NAD^+^) dependent and highly conserved in various systems. They are also the principal regulators of the lifespan in lower life organisms because of their role in controlling reactive oxygen species (ROS) [1]. Silencing the information regulator 2 or Sir2 was the original member of this family, which was first observed in Saccharomyces cerevisiae [2,3,4,5,6]. In a given life form, Sir2 prolongs life by suppressing formation of extra-chromosomal ribosomal DNA circles, which are toxic in yeast [7,8,9,10,11]. In mammals, there are seven different sirtuins. These are SIRT1 through SIRT7. These have a broad range of functions in the cell with respect to energy balance, stress resistance to the cells, genomic stability, and aging [12,13,14,15]. However, not all sirtuin family members are found in the same place. SIRT1 and SIRT2 are found in the nucleus as well as the cytosol, SIRT3, SIRT4, and SIRT5 in the mitochondria and SIRT6 and SIRT7 in the nucleus [16,17,18,19]. This review is primarily concerned with SIRT6. SIRT6 is a protein involved in the regulating chromatin and has been shown to have a number of roles in metabolism, aging, and disease. It could potentially be a useful target in treating several human diseases [20,21,22,23,24]. SIRT6 being tightly bound to chromatin can be described as a NAD^+^ dependent deacetylase concerned with H3K9 and H3K56 (Histone H3 lysine 9 and H3 lysine 56, respectively) [25,26]. The initial uncovering of this histone deacetylation lead to the discovery of roles of these enzymes in variegated processes such as telomere maintenance, DNA repair, and gene expression [27]. The reasoning behind this is that this process is related to a less accessible chromatin that also has a conformation, which is closed and, therefore, was less pellucid [28,29,30,31,32].

### 1.1. Structure of SIRT6 and its Activity

**Figure 1:**
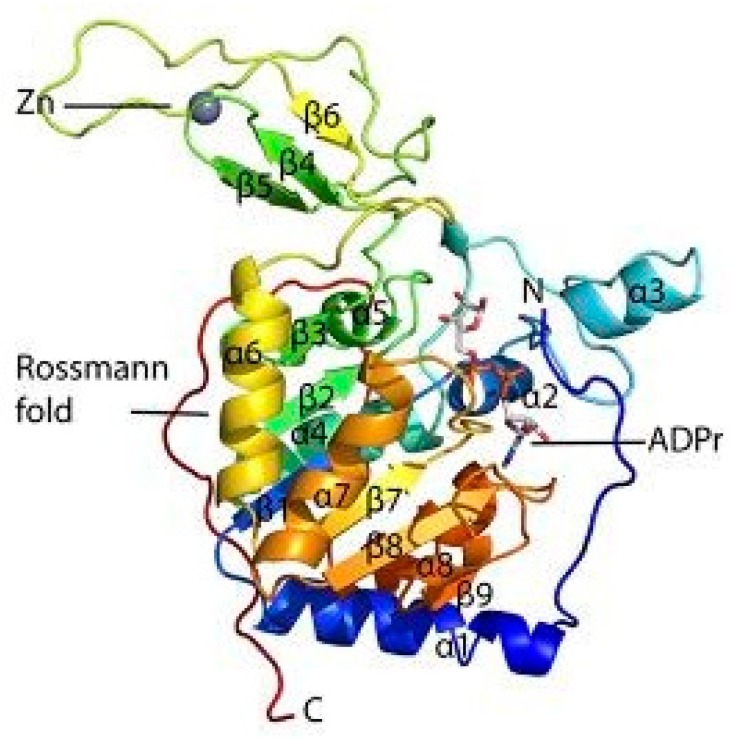
Structure of a SIRT6 monomer (adapted from reference [21]).

The catalytic core region of the sirtuin family of proteins include approximately 275 amino acids. Their length and sequence fluctuate due to the variable N-terminal extensions (NTE) and C-terminal extensions (CTE) they possess. Further catalysis and regulation of sirtuins is promoted by the presence of large and structurally homologous Rossmann-fold domain for NAD^+^ binding. In addition, a more structurally assorted, zinc-binding domain also exists within the catalytic core region [33,34,35,36,37]. The structural monomer of SIRT6 is shown below in Figure 1 [38]. The human SIRT6 can be best distinguished as a NAD^+^-dependent histone deacetylase that contains 355 amino acids. Lysine is deacetylated through the coupling of SIRT6 with NAD^+^ hydrolysis yielding *O*-acetyl-ADP (adenosine 5′-diphosphoribose), nicotinamide, and a deacetylated substrate. Contrary to all other sirtuins, SIRT6 can bind NAD^+^ in the absence of an acetylated substrate. This enables SIRT6 to act as an NAD^+^ sensor while the nicotinamide produced inhibits SIRT6 activity. SIRT6 occupies an open conformation where the zinc-binding motif is separated from the Rossman-fold domain. The presence of hydrogen bonds between the Rossman-fold and the zinc-binding motif stabilizes the structural conformation of SIRT6 [38,39,40,41,42]. Recently, it has been discovered that the free fatty acids (FFAs) endogenously activates SIRT6 deacetylase in vitro. However, it still remains to be seen how it impacts the deacetylase activity in vivo [43,44,45,46,47]. The deacetylase activity of purified protein in vitro is 1000-fold lower for SIRT6 when compared to SIRT1. SIRT6 has also been known to remove long-chain fatty acyl groups from lysine residues in addition to removing the single acetyl groups. In addition to the deacetylation reaction, SIRT6 also used NAD^+^ to produce *O*-myristoyl-ADP, the deacetylated substrate, and nicotinamide. This demyristoylation activity is about 300 times higher than the SIRT6 in vitro deacetylation activity [48]. Furthermore, SIRT6 can use NAD^+^ as a substrate with poly-(adenosine diphosphate-ribose) polymerase 1 (PARP1) and itself, which depicts a very weak ADP-ribosylation activity [49]. A thorough characterization of the CTE and NTE of SIRT6 unravels further important functional roles that they play in biological systems. In order to facilitate proper sub-cellular targeting, the CTE of SIRT6 contains the nuclear localization signal 345 PKRVKAK 351 that is expendable for enzymatic activity. In contrast, the NTE of SIRT6 is climacteric to the intrinsic H3K9 and H3K56 deacetylase activity in cells and also in chromatin association. Absence of the NTE significantly decreases the deacetylase activity of SIRT6 through defective enzymatic activity. Furthermore, the NTE and CTE of SIRT6 are of significance for nucleosome binding along with its diverse enzymatic activities [50].

### 1.2. Observed Implications of SIRT6 

In the cellular domain, a deficiency in SIRT6 may lead to several alterations in glucose metabolism, genomic stability, sensitivity to radiation, and hydrogen peroxide [29]. Practical evidence of this is seen in murine models. When mice are deprived of SIRT6, they exhibit phenotypes along the lines of shortened life expectancy, cancer, and metabolic disorders. In direct contrast, mice that have an overexpression of SIRT6 are seen to have an increase in life expectancy. For example, mice that are put on a specific diet regimen, which is calorie-restricted, are observed to overexpress SIRT6 that may offer protection against many age-related illnesses [20,21,22,23,24]. SIRT6-deficient mice have also shown signs of hypoglycemia, loss of subcutaneous fat, curved spines, and diminished levels of insulin growth factor-1 (IGF-1) [28].

The focus of this review will be on the advances made in understanding of SIRT6 and its implications on aging and several major human diseases. Lastly, we will relate recent findings to possible future avenues of research that could be explored by researchers in order to make advances with regards to SIRT6 and potential therapy benefits it may offer.

## 2. Role of SIRT6 in DNA Repair and Aging-Related Processes

**Figure 2:**
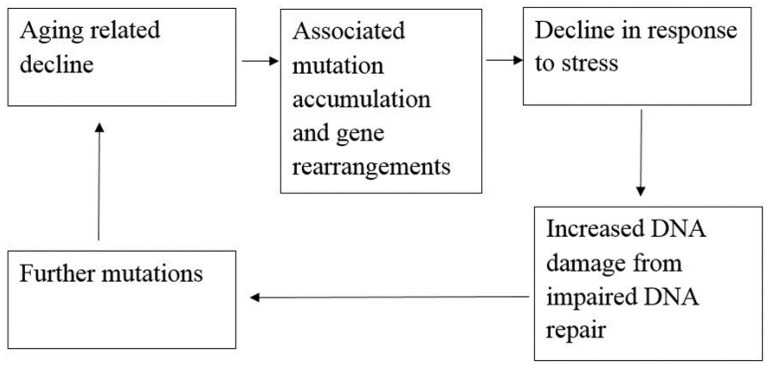
The cycle of aging-related decline and genetic instability (adapted from reference [56]).

Even though aging-related physiologic decline and increased human mortality is very poorly understood in biology, genomic stability-related studies on SIRT6 may offer some useful insight into the arena. The cycle that aging has with genomic instability and DNA damage presents a very critical problem shown in Figure 2. Since DNA is a critical target for aging related issues, the involvement of SIRT6 in DNA repair requires some attention. Especially, in processes such as maintenance of telomeres, repairing of double-strand breaks and break excision repair [51,52,53,54,55].

### 2.1. Maintenance of Telomeres

SIRT6 deficiency leads to several problems such as random telomere sequence loss that is replication associated, accumulation of DNA damage, and chromosomal end-to-end fusion. These lead to genomic instability and may cause the early death of cells. In the case of telomere maintenance, the deacetylation of H3K9 and H3K56 during the S-phase is a key process that is required for the association of Werner Syndrome Protein (WRN) and telomeric chromatin [56,57,58,59,60,61]. WRN is a major player in the genome stability in general. It is crucial in processes such as DNA replication, telomere metabolism etc. This protein may have a part to play in the replication of lagging telomeric DNA as well as the correct capping of telomeres. The importance of the deacetylation process in this case indicates the genomic instability associated with SIRT6 deficiency, which may be explained by the association between chromatin and WRN [62,63]. Mammalian telomeres consist of TTAGGG repetitive sequences that terminate in a three single-stranded Grich overhang. The three single-stranded overhang invades a duplex region of the telomere to form a single-stranded displacement (D) loop, which folds the telomeres into a structure termed the t-loop. The enzyme telomerase, which is a specialized ribonucleoprotein complex containing an RNA template (Terc) and a reverse transcriptase catalytic subunit (Tert), is primarily responsible for the maintenance of telomeres [57,59,64,65].

### 2.2. Base Excision Repair

SIRT6 may also have a role to play in base excision repair. After a number of studies done on knockout mouse models, researchers have hypothesized that SIRT6 may be fostering base excision repair. Even though more experimental evidence is still required before one could make more definitive conclusions, but a few likely explanations of the mechanisms have been posited. SIRT6 deficiency is functionally linked to base excision repair (BER) and compromises the BER pathway that repairs the spontaneously occurring single-stranded DNA lesions [20]. One possibility is that SIRT6 may be regulating chromatin as a way to increase DNA accessibility to factors that lead to base excision repair. Another possibility is that increased levels of SIRT6 may be associated with decreased oxidative stress. This may be due to the role SIRT6 holds in the activation of PARP1 [29].

### 2.3. Double Strand DNA Break Repair

There is also some evidence regarding the role of SIRT6 in double-stranded DNA break repair. Increased levels of SIRT6 has been associated with improved homologous recombination and non-homologous end joining. In addition, some SIRT6 activities such as deacetylation and mono-ADP-ribosylation are required in the DNA repair process. In human somatic cells, telomerase is limited and, therefore, results in progressive shortening of the telomeres since DNA polymerases is unable to fully replicate the extreme terminus of the lagging DNA strand. Therefore, telomere attenuation occurs with each round of DNA replication and is eventually critically shortened [20,29,66]. The interaction that SIRT6 has with PARP1 not only plays a role in base excision repair but double strand repair as well. Evidence suggests that this interaction is only significant when there is oxidative stress involved in the case of double strand repair [49]. Studies involving SIRT6 substrate CtIP (C-terminal binding protein (CtBP) interacting protein) have revealed that SIRT6 loss is associated with accumulated DNA damage, reduced rates of homologous recombination, and increased cell exposure to agents that induce double strand breaks [67,68]. Moreover, SIRT6 has been observed to interact with SNF2H (SWItch/Sucrose non-fermentable-related matrix-associated actin-dependent regulator of chromatin) in order to facilitate the expression of SNF2H in double strand DNA damage sites. Both in vitro and in vivo evidence suggests that SIRT6 histone deacetylation and the interaction with SNF2H both play a crucial role in DNA damage repair mechanisms [69]. A list of identified substrates of SIRT6 is shown on Table 1.

### 2.4. Aging and Life Expectancy

Only very recently, studies involving transgenic mice revealed the role that SIRT6 plays in life expectancy [71,72,73,74,75,76,77]. Particularly in male transgenic mice, an overexpression of SIRT6 was correlated with a 15% increase in life expectancy. A potential explanation for this phenomena was the reduction in insulin-like growth factor signaling in adipose tissue [71]. Stress granules (SGs), which are RNA/protein complexes that are formed in response to stress on the cell, are important in prolonging life and are usually impaired with age and aging-related processes. Studies have shown that SIRT6 may localize to SGs in the cytoplasm in response to stress and aid in recovery from stress that may arise from oxidative damage, heat shock, or deprivation of nutrients [78,79]. Therefore, a loss of SIRT6 may be associated with the disruption of these SGs and the acceleration of aging-related processes. In addition to its role as a histone deacetylase, it would not be entirely implausible for SIRT6 to be a cytoplasmic regulator of SGs, which affects life expectancy. This, is conjunction with its linked roles in metabolism, heart disease, genetic stability, and cancer may make SIRT6 a crucial player in human aging [80,81,82,83].

## 3. SIRT6 in Glucose Metabolism and Diabetes

Diabetes is a major human disease that is characterized by irregularities in glucose regulation and SIRT6 has been demonstrated to be a principal regulator of glucose homeostasis. Diabetes is often caused by β-cell dysfunction and recent studies have shown that SIRT6 may be important in glucose stimulated insulin secretion from these pancreatic β cells and SIRT6 may help improve insulin secretion in diabetics [84]. In knockout mouse models, mice that are deficient in SIRT6 tend to show extreme hypoglycemia, which causes premature death [16]. When inspecting the cause of this phenotype, the primordial causes such as intestinal glucose uptake or increased secretion in the kidneys were overshadowed by an increase in uptake in adipose and muscle tissue. Moreover, in vitro and in vivo studies with multiple cell models have revealed that this increase in uptake of glucose may specifically be related to SIRT6 deficiency [85].

### 3.1. SIRT6 in Glycolysis and the Suppression of Glucose-Metabolic Genes

Since this glucose uptake in adipose and muscle tissue falls on the domain of irregularity, it is necessary to inspect why this is so. To be specific, SIRT6 is responsible for suppressing a few genes that can be characterized as glucose-metabolic by interacting with HIF1α (hypoxia inducible factor-1α) target genes. These glucose-metabolic genes were lactate dehydrogenase (LDH), pyruvate dehydrogenase kinase 1 (PDK1), and glucose transporter-1 (GLUT-1). HIF1α has a key role in this suppression process since it is known to coordinate several genes that activate processes such as glycolysis [85]. Under nutrient stressed conditions or hypoxia, glycolysis plays an important part by taking over the metabolic role. Therefore, researchers have concluded reasonably that SIRT6, which is a negative regulator of enhanced glycolysis, is responsible for the repression of HIF1α and eventually leads to a decrease in cellular glucose uptake [86].

### 3.2. SIRT6 in Gluconeogenesis 

Researchers have also determined the role SIRT6 plays with regards to expression of gluconeogenesis genes. Studies show that these expressions were higher in livers that were deficient in SIRT6. This posits the theory that the liver may be trying to compensate for the onset of hypoglycemia that results from this lower expression of SIRT6 [87]. Peroxisome proliferator-activated receptor-α coactivator 1α (PGC-1α) is a principal regulator that is responsible for stimulating gluconeogenesis in the liver. It does so by increasing the level of gluconeogenesis enzymes that lead to more glucose uptake in the cells. The interaction between SIRT6, PGC-1α, and a protein named general control non-repressed protein 5 (GCN5) leads SIRT6 to reduce the amount of glucose production. Conversely, a lower expression of SIRT6 leads to a higher amount of glucose production [88]. In another study, wild type mice were compared to liver specified Forkhead box O1 (FOXO1) knockout mice with respect to SIRT6. It was observed that the overexpression of SIRT6 reduced gluconeogenesis expression in the wild type but not in the FOXO1 mice. Therefore, researchers posited the theory that SIRT6 is responsible for regulating gluconeogenesis in the liver by modulating both PGC-1α and FOXO1 even though more research has to be done to further clarify the issue [89].

## 4. Pharmacological Intervention with Small Molecule SIRT6 Inhibitors for SIRT Associated Ailments

Since the involvement of SIRT6 holds such promise, researchers are in the process of identifying SIRT6-targeted therapeutic agents that may have a wide range of uses in diabetes as well as several other diseases. Table 2 shows a few relatively new SIRT6 inhibitors and their corresponding Asinex IDs that have shown some marked inhibition of SIRT6 [90]. In 2017, Sociali et al. studied the pharmacological effects of one of these compounds called 2,4-dioxo-*N*-(4-(pyridin-3-yloxy)phenyl)-1,2,3,4-tetrahydroquinazoline-6-sulfonamide (Asinex ID SYN17739303 in Table 2) on a mouse model for Type 2 diabetes mellitus. The mice were six weeks old and were fed a high-fat diet. This compound was administered for 10 days and results indicated an improvement in glucose regulation via the oral glucose tolerance test (OGTT), which led the researchers to conclude that small molecule inhibitors of SIRT6 may be a functional strategy in improving glycemic control for Type 2 diabetics. In addition to these positive findings, there was also a notable increase in glucose transporters as well as reduced levels of insulin, triglycerides, and cholesterol observed in the same study possibly paving the way for small molecule inhibition of SIRT6 for other diseases as well [91]. Further pharmacological interventions include Rosiglitazone (RGZ), which is an antidiabetic agent that increases Sirt1/6 expression and its activity in rat livers in vivo. In a rat model of moderate obesity and insulin resistance and a cell model of hepatocyte steatosis, studies have concluded that RGZ significantly reduced lipid accumulation and activated the Sirt1/6-LKB1-AMPK pathway. It has been also observed that Sirt6 knockdown inhibited the protective effects of RGZ [85,92]. SIRT6 might be a novel and attractive target for developing future therapeutics for treating aggressive human neuroblastoma (NB). Studies have also elucidated the role of SIRT6 in differentiation and proliferation of NB where BE(2)-C cells were treated with nicotinamide (NAM), which is a non-specific SIRT inhibitor. It was observed that SIRT6 knockdown in BE(2)-C cells resulted in a reduction of cell proliferation, which corresponds with induction of p21CIP1 expression and the G1 cell-cycle arrest [56,57,58,93,94]. SIRT6 expression was reduced in differentiated human NB sections and RA-induced differentiation in BE(2)-C cells [60,94].

## 5. SIRT6 in Heart Disease

**Figure 3:**
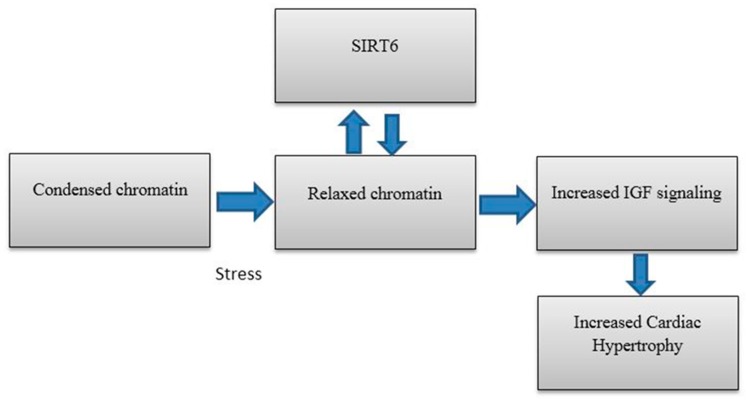
During normal function, SIRT6 is responsible for inhibiting the expression of Insulin-like growth factor 1 (IGF) signaling genes. Under stressed conditions, however, SIRT6 in cardiac tissue is reduced leading to an increase in IGF signaling and an increase in cardiac hypertrophy and multiple complications (adapted from reference [96]).

Researchers have also identified results that highlight the importance of SIRT6 in cardiac failure and hypertrophy. Nicotinamide adenine dinucleotide (NAD) dependent deacetylases are critical in case of hypertrophy in cardiomyocytes [95]. In 2012, Cai et al. discovered that Nicotinamide mononucleotide adenylyltransferase 2 (Nmnat2) is a key enzyme in NAD biosynthetic pathway and its activity and that protein expression were reduced in the case of cardiac hypertrophy[95]. Therefore, angiotensin II (Ang II) induced cardiac hypertrophy was seen to be reduced when Nmnat2 was overexpressed in mouse models. There was a positive correlation observed when the relative SIRT6 activity (protein expression and enzyme activity) was measured with regards to cardiomyocytes that were transfected with Nmnat2 over time. Therefore, it becomes reasonable to conclude that the activation of SIRT6 in Ang-II induced hypertrophy may have been related to Nmnat2 expression and SIRT6, which, in this case, acts as a negative regulator in the case of cardiac hypertrophy. The fact that SIRT6 is a negative regulator of cardiac hypertrophy, which is further demonstrated in another study where SIRT6 deficient mice were compared to SIRT6 transgenic mice and the latter showed attenuated cardiac hypertrophy. In this particular study, it is shown that SIRT6 directly inhibits IGF signaling and inhibition of IGF signaling leads to a reduced rate of cardiac hypertrophy and vice versa [91]. This is illustrated below in Figure 3.

More recently, in 2016, Lu et al. demonstrated the importance of SIRT6 in case of cardiac hypertrophy when they tested the autophagy activity of cardiomyocytes under isoproterenol treatment. Since reduced autophagy was seen to contribute towards the pathogenesis of cardiac hypertrophy, it was demonstrated in this study that the increase in the levels of SIRT6 lead to an enhanced autophagy of cardiomyocytes. SIRT6 was shown to have protective effects against cardiac hypertrophy with the onset of autophagy by promoting the transcription factor FOXO1 (also plays a role in gluconeogenesis) [97]. From the data currently available, it would seem that Nmnat2 manipulation, IGF signaling modulation and cellular autophagy targeting may be useful in targeting SIRT6 for future drugs in order to treat cardiac hypertrophy.

## 6. SIRT6 and Cancer

Considering the wide range of processes that SIRT6 seems to be potentially involved in, it comes as no surprise that it may also be linked to cancer progression and tumor growth.

### 6.1. Tumor Growth Resulting from Altered Glycolysis

In terms of cell proliferation, tumor cells have specific and often exceptional metabolic requirements that are necessary for cell division and growth. In order to proliferate, nutrient uptake of these cells in a cell-autonomous fashion and re-organization of certain pathways (specifically, metabolic pathways) may be necessary to engender the biosynthesis of macromolecules that are required for this process [98]. One of the best possible explanations of this re-organization of metabolic pathways was given by Otto Warburg in 1927 who posited the Warburg effect [99]. Warburg observed that the enhancement of glycolysis under the presence of excess oxygen (aerobic condition) was responsible for this reprogramming of cancer cells. At this point, it is worthwhile to come back to the modulation of HIF1α by SIRT6. As mentioned earlier, the activation of HIF1α (that corresponds to a deficiency in SIRT6), which is a transcription factor, may lead to enhanced glycolysis and an increase in the uptake of glucose in the cell [85]. This phenomenon is analogous to that of enhanced glycolysis that takes place in tumor cells. The study of this phenomena in mouse embryonic fibroblast cell lines showed that tumor growth was possible without the activation of oncogenes and only under enhanced glycolysis [86]. In vivo studies further corroborated this fact when genetic analysis and fluorodeoxyglucose positron emission tomography (FDG-PET) with SIRT6 deficient knockout mouse models revealed that the mice were three times as prone to acquire adenomas when compared to wild-type mice. When comparing mouse models for colorectal carcinomas and pancreatic ductal adenocarcinomas with wild type mice, it was seen that the HIF1α target genes i.e., LDH, GLUT1, and Phosphofructokinase-1 (PFK1) were noticeably unregulated. Consistent inferences were made when studying the survival rates of colorectal cancer as well. SIRT6 deficient mice were three times more likely to relapse in cancer progression when compared to mice that showed high levels of SIRT6. All of these results taken together demonstrate the importance of SIRT6 in tumor progression [86,100,101,102,103,104]. An important avenue for treatment was explored when the administration of dichloroacetate (DCA) was seen to slow down tumor formation of these SIRT6 deficient mice by inhibiting PDK1, which is also a key target gene for HIF1α [86].

### 6.2. SIRT6 and the Initiation of Hepatic Cancer

In vivo studies have also revealed the importance of up-regulation of SIRT6 at the early stages of hepatic cancer. In this case, the primary focus was c-JUN and c-FOS, which are the components of the transcription factor AP-1 [105,106]. From a mechanistic standpoint, c-JUN and c-FOS are responsible for increasing the levels of SIRT6 expression and then SIRT6 in turn represses the activity of BIRC5 (also known as survivin). In other words, SIRT6 is a negative regulator of BIRC5. Targeting BIRC5’s anti-apoptotic activity may be used to slow down cancer development at the early stages of hepatic cancer. A vital development in this case was the identification of the regulation pattern between c-JUN, c-FOS, SIRT6, and BIRC5 in dysplastic liver nodules. Even though further studies may be warranted in this case, this pivotal knowledge about SIRT6 up-regulation may prove to be very useful when combating tumorigenesis in the liver or premalignant liver lesions at the early stages of cancer development. However, this pathway does not function at advanced stages of hepato-cellular carcinoma [105].

### 6.3. Aberrant Behavior of SIRT6 in Other Forms of Cancer

In the aforementioned cancers, a trend was seen that the up-regulation of SIRT6 was associated with generally beneficial effects. However, this is not the case for all forms of cancers across the board. In the case of squamous cell carcinoma, in vitro and in vivo studies have demonstrated that high levels of SIRT6 are expressed because of the down-regulation of RNA-34a (miR-34a) [106]. In another study, with chronic lymphocytic leukemia (CLL) patients, it was discovered that the patients exhibited four times as high levels of SIRT6 compared to control groups leading the researchers to conclude that the overexpression of SIRT6 may be associated with a poor prognosis for CLL patients [107]. The biological relevance of SIRT6 in acute myeloid leukemia (AML) includes frequent up-regulation in tumor cells compared with normal CD34+hematopoietic progenitors. SIRT6 loss unleashes genomic instability and consequently triggers hypersensitivity to clinically used DNA-damaging agents such as daunorubicin (DNR) and cytarabine (ARA-C) [107,108]. Studies have depicted that hematologic cancers including AML have constitutive ongoing DNA damage as well as a steadily activated DNA repair response and, therefore, strategies including high DNA damage and reduced DNA repair by SIRT6 inhibition has the potential to decrease tumor growth and may benefit patients with otherwise unfavorable outcomes [107,108,109].

The study of SIRT6 in relation to cancer raises more questions than actual answers. Researchers have to work diligently to attempt to answer these questions in the future. Unfortunately, at this stage, it is difficult to make definitive conclusions about the extent of insolvent of SIRT6 on cancer progression and tumor growth. It is especially interesting that overexpression of SIRT6 may offer benefits by protecting against genomic instability in some cases but may act as an oncogene in other cases or it may be doing both in all of the cases. Regardless, the role of SIRT6 in caner still remains highly complex.

## 7. SIRT6 in Neurodegenerative Diseases and Brain Aging

More recently, SIRT6 has also been shown to have implications in brain aging and major neurodegenerative diseases such as Alzheimer’s disease (AD). In a recent study, cellular localizations were studied in the cerebral cortex and hippocampus of 24-month-old mice and 3-month-old mice. It was observed that SIRT6 expression was lower in the older mice [110,111]. In 2016, Jung et al. reported two critical observations that demonstrated its role in AD patients. The group observed these findings in three different in vitro and in vivo models including the HT22 mouse hippocampal neurons, brains of AD patients, and brains of 5XFAD AD mice. The first critical observation was that Aβ42, which is a significant component of aged plaques, were inducing DNA damage that would otherwise be prevented in the HT22 mice by an overexpression of SIRT6. The second observation was that Aβ42 decreased SIRT6 expression overall in all of the three models [112]. In 2017, Kaluski et al. showed that severely reduced levels of SIRT6 may incite neurodegeneration of Alzheimer’s patients by promoting DNA damage, cell death, and hyper phosphorylation of tau proteins, which are abundant in the central nervous system and neurons [113]. All of this put together shows that there may be a strong link between SIRT6 and neurodegenerative diseases. In a study published as recently as 2017 in *Neuroscience,* studies on SIRT6 showed that it may be responsible for protecting the brain from cerebrovascular ischemia and may be identified as a potential therapeutic target for ischemic stroke [114]. It is obvious that more research in this area is warranted. However, for now, it may be reasonable to infer that SIRT6 may be of paramount interest in neuroscience in the near future.

## 8. Advances Made with SIRT6 in Other Areas

In addition to glucose metabolism, cancer, and aging related processes, SIRT6 has also been shown to be a negative regulator of triglyceride synthesis and affect liver disease. Its deficiency has resulted in the accumulation of triglycerides that may lead to fatty liver disease [115]. Recent studies have also demonstrated the importance of SIRT6 in cholesterol homeostasis by studying its regulation patterns with respect to the protein convertase subtilisin/kexin type 9 (PCSK9) gene. In this case, knockout mouse models have shown that PCSK9 deficient mice exhibited lower levels of LDL that correspond to an overexpression of SIRT6 [116]. SIRT6 has also been shown to have pro-inflammatory and anti-inflammatory roles depending on the type of cell that is involved [117,118]. Studies have depicted that SIRT6 promotes inflammation by enhancing tumor necrosis factor α (TNF) expression. A study conducted by Bauer et al. showed that SIRT6 enhanced the expression of pro-inflammatory cyto-/chemokines such as interleukin-8 (IL8) and TNF, which promoted cell migration in pancreatic cancer cells through enhanced Ca^2+^ responses [119]. SIRT6 also increased the intracellular levels of ADP-ribose through its enzymatic activity. Conversely, the transient receptor potential cation channel, subfamily M, member 2 (TRPM2), and Ca^2+^ are shown to be involved in the expression of SIRT6-induced TNF and IL8. It was also observed that SIRT6 increased the nuclear levels of the Ca^2+^-dependent transcription factor, nuclear factor of activated T cells (NFAT), and cyclosporin A. These results further confirmed the instrumental role for SIRT6 in the expression of pro-inflammatory, pro-angiogenic, and chemotactic cytokines [117,118,119,120]. Sugatani et al. in Bone demonstrated that SIRT6 deficient mice also showed characteristics of osteopenia leading the researchers to conclude that molecular mechanisms of SIRT6 in the case of bones could lead to potential therapeutic targets that could reverse age-related bone loss [121]. Another study conducted by Rahnasto-Rilla et al. has shown that flavonoids modulate the activity of SIRT6 due to its implicated role in longevity, metabolism, DNA-repair, and inflammatory response reduction. Catechin derivatives with galloyl moiety have demonstrated significant inhibition potency against SIRT6 at 10 µM concentration. Cyanidin, which is the most potent SIRT6 activator, has been shown to produce a 55-fold increase in SIRT6 activity compared to the 3–fold to 10-fold increase for the others. In addition, in Caco-2 cells, SIRT6 expression has been significantly increased by cyanidin [122]. Furthermore, Kokkonen et al. examined the capability of SIRT6 to deacetylate a set of five fluorogenic substrates based on p53 and histone H3 sequences. The substrate designed around H3K56 deacetylation site exhibited the best signal-to-background ratio and was selected. It was observed that EX-527, quercetin, and three pseudo-peptidic compounds were the most potent SIRT6 inhibitors since they exhibited over 50% deacetylation inhibition [123]. These are just a few examples of the numerous roles SIRT6 may play in different processes and it would seem that there is still significant work to be done in this area.

## 9. Conclusions

It is clear that, with the emergence of these new studies on SIRT6, the substance has managed to pique the curiosity of a number of researchers in the biomedical sciences. The role of SIRT6 as a regulator or even a nutrient detector in cells has diversified its impact on aging-related processes and major human diseases such as cancer, diabetes, neurodegenerative diseases, and heart disease. SIRT6 has been shown to be involved in gene expression in the nucleus with regards to chromatin and, more recently, has also been shown to take part SG formation in the cytosol. For now, it would seem that the pleiotropic effects of SIRT6 is clear. However, the extent of involvement in each individual process still remains hazy. The variegated roles of SIRT6 may expand even further beyond what is currently known but only time will reveal its true effects on human biology and various diseases. Even its implications in neuroscience could lead to potential solutions to long-standing problems such as Alzheimer’s disease. Evidently, SIRT6 needs to be studied further. More time and resources are mandated in order to understand and identify potential therapeutic targets in processes such as glycolysis, glycolysis, tumorigenesis, osteoblastogenesis, and more. More studies on SIRT6 may possibly eventuate strong therapeutic targets and it may be possible to use rational drug design in order to alleviate or possibly even cure some primordial diseases that have proven hard to eliminate.

## Figures and Tables

**Table 1:** A few substrates of SIRT6 and their linked functions in cell-related to aging (adapted from reference [70]).

Substrate	Linked Functions in Cell
H3K9ac	Regulation of transcription, stability of telomeres, response to DNA damage
H3K56ac	Regulation of transcription, stability of telomeres, response to DNA damage
H3K18ac	Silencing of heterochromatin
NPM1	Cellular Senescence
PARP1	DNA double-strand break repair and base-excision repair
KAP1	DNA double-strand break repair

H3K9ac = Histone H3 lysine 9 acetylation, H3K56ac = Histone H3 lysine 56 acetylation, H3K18ac = Histone H3 lysine 18 acetylation, NPM1 = Nucleophosmin 1, PARP1 = poly-(adenosine diphosphate-ribose) polymerase 1, KAP1 = The Krüppel associated box (KRAB)-associated protein-1.

**Table 2:** Small molecule SIRT6 inhibitors (as reported by Parenti et al. [90]).

Asinex ID	Compound Structure	Percentage Inhibition of SIRT6 at 200 µM Concentration of the Compound
SYN17739303	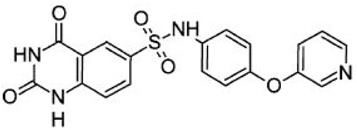	100 ± 4
BAS13555470	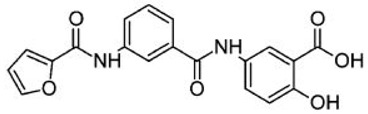	62 ± 7
SYN10366754	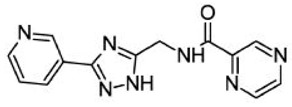	12 ± 3
BAS00417531	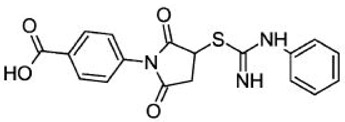	66 ± 6
